# The COVID-19 lockdown: Impact on the mental-wellbeing of out-patients with chronic medical conditions in a teaching hospital in nigeria

**DOI:** 10.1192/j.eurpsy.2021.281

**Published:** 2021-08-13

**Authors:** A.J. Ogunmodede, O. Buhari, J. Ogunmodede

**Affiliations:** 1 Dept Of Behavioural Sciences, UNIVERSITY OF ILORIN TEACHING HOSPITAL, ILORIN, Nigeria; 2 Dept Of Behavioural Sciences, UNIVERSITY OF ILORIN & UNIVERSITY OF ILORIN TEACHING HOSPITAL, ILORIN, Nigeria; 3 Dept Of Medicine, UNIVERSITY OF ILORIN & UNIVERSITY OF ILORIN TEACHING HOSPITAL, ILORIN, Nigeria

**Keywords:** COVID-19, LOCKDOWN RESTRICTION, MENTAL WELLBEING

## Abstract

**Introduction:**

Lock-down restrictions were introduced in most countries of the world at the onset COVID-19 pandemic. It was associated with serious implications for healthcare delivery, with affectation of access to medical services for patients with chronic medical conditions. It is important to assess the impact of this on the subjective feeling of mental wellbeing in these patients.

**Objectives:**

This study aimed to assess the access to health care services during the lock-down as well the perceived affectation of the mental, physical and social wellbeing and their related factors in patients with chronic illnesses in Ilorin.

**Methods:**

This study was a cross-sectional study, involving 166 patients being managed for different chronic medical conditions, attending the Medical Outpatient Clinics of the University of Ilorin Teaching Hospital. A socio-demographic questionnaire and a structured questionnaire designed by the researchers was used.

**Results:**

The mean age of all respondents was 49.5+18.5. 25.3% of respondents were being managed for heart-related conditions. 54 respondents(32.5%) reported a negative affectation of their mental wellbeing, which included changes in mood, sleep pattern and feelings of being overwhelmed and unable to cope properly. The age (p= 0.031) and employment status(p=0.015)of the patient were significantly associated with a subjective feeling of negative affectation of wellbeing.

**Conclusions:**

The impact of the COVID-19 pandemic lockdown on the mental well-being of patients with chronic medical conditions is significant and calls for a more strategic plan for delivery of health care services during pandemic situations with focus on the mental well being of patients.

**Disclosure:**

No significant relationships.

